# A Review of Passive RFID Tag Antenna-Based Sensors and Systems for Structural Health Monitoring Applications

**DOI:** 10.3390/s17020265

**Published:** 2017-01-29

**Authors:** Jun Zhang, Gui Yun Tian, Adi M. J. Marindra, Ali Imam Sunny, Ao Bo Zhao

**Affiliations:** 1 School of Electrical and Electronic Engineering, Newcastle University, Newcastle upon Tyne NE1 7RU, UK; zhangj89sysu@gmail.com (J.Z.); A.M.J.Marindra2@newcastle.ac.uk (A.M.J.M.); a.sunny@newcastle.ac.uk (A.I.S.); a.zhao@newcastle.ac.uk (A.B.Z.); 2 School of Automation Engineering, University of Electronic Science and Technology of China, Chengdu 611731, China

**Keywords:** structural health monitoring (SHM), radio frequency identification (RFID), passive sensors, antenna, strain, crack, corrosion

## Abstract

In recent few years, the antenna and sensor communities have witnessed a considerable integration of radio frequency identification (RFID) tag antennas and sensors because of the impetus provided by internet of things (IoT) and cyber-physical systems (CPS). Such types of sensor can find potential applications in structural health monitoring (SHM) because of their passive, wireless, simple, compact size, and multimodal nature, particular in large scale infrastructures during their lifecycle. The big data from these ubiquitous sensors are expected to generate a big impact for intelligent monitoring. A remarkable number of scientific papers demonstrate the possibility that objects can be remotely tracked and intelligently monitored for their physical/chemical/mechanical properties and environment conditions. Most of the work focuses on antenna design, and significant information has been generated to demonstrate feasibilities. Further information is needed to gain deep understanding of the passive RFID antenna sensor systems in order to make them reliable and practical. Nevertheless, this information is scattered over much literature. This paper is to comprehensively summarize and clearly highlight the challenges and state-of-the-art methods of passive RFID antenna sensors and systems in terms of sensing and communication from system point of view. Future trends are also discussed. The future research and development in UK are suggested as well.

## 1. Introduction

The high costs and liabilities associated with potential failures have made structural health monitoring (SHM) an integral and necessary security measure to ensure safe and reliable operation of large-scale structures, e.g., railway, pipelines, dams, bridges, and aircrafts. While these structures are designed to ensure that they operate safely under anticipated loading scenarios, deterioration and damage can occur over their operational lifespan [1]. In particular, repeated exposure to operational and environmental loads over decades of service will inevitably introduce deterioration such as corrosion and fatigue. For example, the last several decades have witnessed unprecedented prosperity in the railway industry globally. The surface of the rail web (cross section connecting the rail head with the foot) and foot (base support of the rail) can be damaged by corrosion, leading to fractures and derailments [2], which will jeopardize the safety. Regardless of the incidence of such failures has been progressively reducing through greater recognition of the potential failure mechanisms, improvements in materials selection, informed system management, etc. [3], these structures require constant inspections to detect and prevent potential structural problems.

Periodic manual inspections, which are primarily visual, are difficult, unreliable, and nearly impossible in situations where the structures are hard to access, for example, harsh environments impede manual monitoring of critical environment data, or defects incur underneath the surface. Many non-destructive testing and evaluation (NDT & E) techniques, such as ultrasonic [4], pulsed eddy current (PEC) [5], and eddy current pulsed thermography (ECPT) [6], were developed for monitoring defects in structures with good resolution, sensitivity, and reliability. However, these techniques are expensive to implement for a large-scale application because of the labor and wiring costs as well as range-limited because of their power and resolution requirements [7]. In addition to significant costs both in time and resources due to the periodic inspections, these techniques might be too cumbersome to continuously monitor the nucleation and growth of potential defects for in-service larger-scale structures.

Distributed sensor-based SHM is an attractive option for monitoring the structural health of these structures, which can transform time-based maintenance into cost-effective condition-based maintenance. Previous methods for deploying large-scale sensor networks involved running long lengths of cabling which would source power and collect data from each individual sensor; while these methods were necessary for some situations where real-time data was required, the cost, installation difficulty, and maintenance rarely justified their use over manual data collection [8]. By eliminating electric wiring from conventional sensors, wireless sensor networks (WSNs) are inexpensive and easier to install, giving us the ability to compile massive amounts of data which can greatly improve our knowledge of the environment surrounding us. This technique makes distributing sensors over a large area and with high density a reality. However, to enable large-scale pervasive sensor networks which collect big data [9], the sensing platform has to be reliable, energy efficient, and extremely low cost to become a viable long-term solution [10].

For potential forthcoming applications, spatial granularity is a key issue. Current wireless sensing applications make use of battery-powered sensors, but these sensors are at least two orders of magnitude more expensive than their simpler passive counterparts, which limits the granularity of their deployment [11]. Furthermore, battery-powered sensors have limited battery life and in turn, pose a long-term environmental risks with the disposal of billions of batteries [12]. Because of their intended massive use, sensors do not need to be extremely sophisticated or precise; however, they must satisfy requirements of low cost and acceptable reliability in order to be deployed at a finer granularity than active precise wireless sensors. The ultimate goal is to design “smart dust motes”, i.e., autonomous sensing, ubiquitous computing, and communication systems small enough to be easily “dispersed in the environment” [13]. This motivates the development of low-cost, wireless, and passive sensors for large-scale infrastructure and big data applications.

In order to enable such a vision, radio frequency identification (RFID) technology can play a strategic role, thanks to its low-cost, wireless, and “sensing-friendly” capabilities [11]. The last decades have witnessed a rapid growth of RFID technology for identification and tracking because of its unique identification (UID). Besides this common usage, an analogue processing of the physical signals related to the reader-tag communication, could permit to achieve much more information about the target without the need for additional electronics or sensors [13]. Enabling the sensing ability into RFID technology can make the system know the state of the real-world objects [14] and seamlessly integrate within the global cyber-physical systems (CPS) and Internet of Things (IoT) [15]. The sensing capabilities provided by RFID tag antennas in the ultra-high frequency (UHF) bands are perhaps an exciting research trend [16], with great applicability to the emerging paradigm of the IoT as a green technology [17]. The key background is a new paradigm of antenna design that merges together the conventional communication issues with more specific requirements about sensitivity to time-varying boundary conditions [13]. The rationale of this idea lies in the clear dependence of the tag’s input impedance and radar cross section (RCS) on the physical and geometrical features of a real target [18].

The RFID technology, which was originally developed for large-scale asset tracking, happens to be a backbone for building low-cost, passive, and large-scale WSNs. This makes deploying massive amounts of sensors possible in reality. Meanwhile, as the RFID platform is widely accepted throughout industry, large-scale WSNs based on RFID technology can be seamlessly integrated into current off-the-shelf RFID systems. For this reason, we seek to highlight this work aimed at enhancing EPC Class 1 Generation 2 (C1G2) standard compliant RFID devices towards the goal of RFID-based sensors and networking. We use the term antenna sensor herein to represent one type of sensor that uses antennas to “sense” the things [19].

The operational principle of antenna sensors mounted on conductive surfaces is similar to pulsed eddy current NDT [20], the conductive loss and penetration depth of which is proportional to the operating frequency. With increased operating frequency, the spatial resolution can be continuously enhanced by a corresponding decrease in wavelength [21] thereby the size of the antenna sensor. Because of magnetic resonant coupling (MRC) in the wireless power transmission (WPT) [22], the read range between RFID tag and reader in low frequency (LF) or high frequency (HF) bands is quite short, e.g., in the range of several centimetres. This is due to evanescent (mode) coupling. Resulting from electromagnetic (EM) coupling (propagation mode), UHF and ultra-wide band (UWB) antennas can be used to increase the communication distance [23].

**Figure 1:**
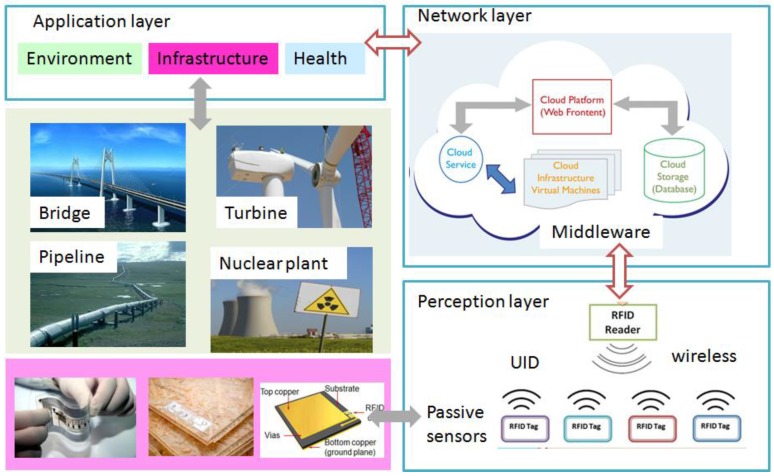
Passive RFID sensor networks for SHM.

A paradigm of RFID based large-scale passive wireless sensor networks for SHM is described in Figure 1. This paper mainly focuses on the UHF band. Some properties of these passive antenna sensors based on RFID technology can be summarized as the following [24,25,26,27]:

- *Simple configuration*: The antenna itself can serve the dual function of communication and sensing. Therefore, no external sensor is needed. For chipless tags, there is even no electronic device. The sensing information is directly encoded into the antenna backscatter behavior. For this reason, the sensor may function in an extreme environment, e.g., high temperature.
- *Passive operation*: The tag chip has its own energy harvesting module, as such, no onboard battery is needed.
- *Medium read range*: The read range for a general passive tag can be up to 10 m, however, the read range largely depends on the frequency, antenna gain, and tag chip’s sensitivity.
- *Low cost:* The cost for each dipole tag is ~$0.10–0.20 for mass production. The antenna sensors can be fabricated on inexpensive substrate materials, such as paper, PVC, using low-cost fabrication techniques, such as inkjet printing.
- *Unique identification*: Each tag has its own UID, which is used to identify the location of the defect as well as connect the things into internet. This sensor multiplexing capability enables densely distributed passive WSNs and parallel interrogation of multiple sensors with anti-collision algorithms.
- *Multimodality*: The antenna can be designed to be sensitive to various physical/mechanical/chemical things in a real-time or periodic.
- *Planar or flexible*: The antenna sensors can be fabricated on low-profile, flexible substrates that completely conform to the surface they attached to.
- *Cover Penetration*: The surface of the metal may be covered with paint, cladding, or a similar compound, and the defect may still be detected because microwaves can penetrate dielectric materials.

The future IoT will consist of heterogeneously connected devices that further extends the borders of the world with physical entities and virtual components [28]. The middleware is designed for the potential integration of a heterogeneous IoT sensor network while the internal data is for seamless access to a Cloud Computing system [29].

The defect information can be extracted by detecting the change in antenna sensors, such as the resonant frequency shift (RFS) from RCS. In addition to mechanical actions, e.g., fatigue, structure (concrete and steel) can develop cracks because of various physical and chemical processes (stress corrosion). Various antenna sensors have been developed for this purpose [30,31,32,33,34,35,36,37,38]. Strain [36,38,39,40,41,42,43,44,45,46] and corrosion [47,48,49,50,51] can also be monitored by antenna sensors, enabling early warnings about structural health. In addition, the liquid level [52] and displacement [53,54] can be monitored as well. Beyond the passive sensors developed for monitoring mechanical/physical parameters, the passive sensors and systems can be expanded for monitoring chemical parameters in the environment with proper electrochemical materials. This is out of scope of this work. Antenna sensors that are sensitive to environmental conditions, e.g., moisture [55,56,57,58], gas [59,60,61], temperature [62,63,64,65,66,67,68], have been demonstrated. More information about this topic can be found in [69,70].

Recent emerging work on passive antenna sensors illustrate the great potential for future SHM in terms of integration of passive sensing, communication, location and identification. Permanent installation removes problems such as signal variability because of texture and geometry changes with position and can provide better damage growth rate estimation by taking data more frequently—at the cost of reduced area coverage; the benefits, however, can only be realized if the systems are *reliable* over long periods, the data obtained can be reliably related to the health of the structure, and any defects are reliably detected with low false alarm rates [71].

Because of the limited scavenging power and fading effect of radio frequency (RF) signal, the challenges for *accurately and reliably* detecting and characterizing defects based on passive antenna sensors in a remote distance are of special concern and need to be systematically studied. In this paper, issues for this type of sensor are outlined. Critical limitations of each issue will be highlighted and potential solutions or alternatives will be explored. To this end, this paper is organized as follows: Section 2 presents the methodology for the literature review and gives an overview of research content and issues involved. The communication issues are presented in Section 3, where the principle and measurable parameters are derived from the backscatter mechanism. The properties of the asymmetric wireless channel and corresponding solutions are also described from communication point of view. Section 4 gives a thorough description of various sensing-oriented issues utilized to make the passive antenna sensors practical. A comprehensive survey of various defect types, antenna sensors, measurement uncertainties, and feature extraction methods related requirements with illustrative examples is presented. This section also discusses some developments of printable technology for flexible, wearable, even chipless applications. Finally, Section 5 summarizes the future research directions.

## 2. Methodology and Categorization

The research methodology employed for examining the adoption of RFID in SHM is a literature review and systematic study. The former can be divided into three phases: literature identification, categorization, and analysis. The latter can be divided into four parts: measurands, antenna sensors, measurement strategy, and feature extraction. Both of them will be respectively described in the following sub-sections.

### 2.1. Methodology

We present here the results of the literature review for past peer-reviewed articles dealing with passive RFID tag antenna sensors and related topics. Articles were collected from the ISI Web of Science with topics (Title, Abstract, and Author Keywords) including sensor or sensing as well as radio frequency identification or RFID. After removing the articles describing location sensing and other irrelevant areas, there were 442 papers on this topic till the end of 2015. It is noteworthy that there were more than 70 papers till the end of September in 2016.

**Figure 2:**
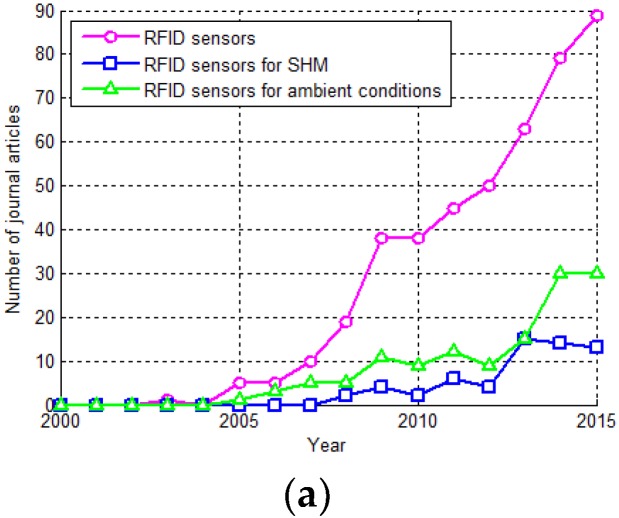
Classification of journal articles for passive RFID tag sensors based on: (**a**) Year; (**b**) Journal; (**c**) Countries/territories (till the end of 2015) and organization in the UK (till Oct. 2016).

First, we highlight the distributions of these articles by year and journal, which are shown in Figure 2a,b. We can find that there are only a few publications up to 2005, but since then, research on passive antenna sensors has grown rapidly. The antenna sensors can be straightforwardly classified into two groups: antenna and sensor. The publications from the *IEEE Sensors Journal* dominate, accounting for more than 10% of the total. The publications can be categorized into countries/territories as seen in Figure 2c. The first four have published more than 80% of the total, while the USA contributes half of these. It is noteworthy the RFID technology is only one way to wirelessly transfer the sensing signal from passive antenna sensors [72]. Therefore, the following analysis is based on but not limited to these articles.

### 2.2. Context and Content

**Figure 3:**
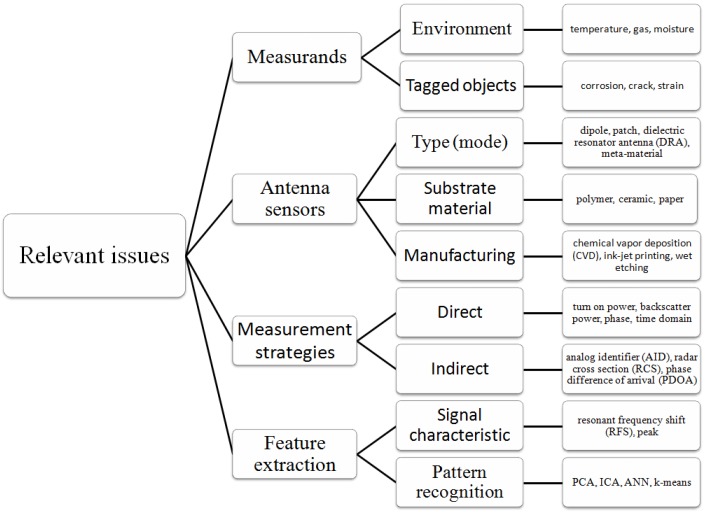
Relevant issues in passive antenna sensors and systems based on RFID technology.

The design and development of passive antenna sensors and systems including direct and indirect sensing through antennas remain a challenging task. The major issue arises because of a tradeoff among sensing and communication, in particular between resolution, sensitivity, size, read range, and robustness. This tradeoff and more relevant issues, as shown in Figure 3, influence the choice of antenna type, sensing principle, substrate material in the tag, implementation of test strategies and selection of sensing variables in the reader, and development of the feature extraction method. Most of them will be covered in the following sections.

## 3. Communication Issues and Solutions

A rigorous characterization of backscattered signals from passive antenna sensors is fundamental for feature extraction with respect to influences from defects and measurement conditions, e.g., wireless interrogation using a reader in a stand-off distance. In this section, we first review the measurable parameters via backscatter communication. Then challenges and possible solutions for the transmission of analogue signal via a wireless channel are discussed in terms of channel model and coherent demodulation from communication point of view.

### 3.1. Backscatter Communication and Measurable Parameters

**Figure 4:**
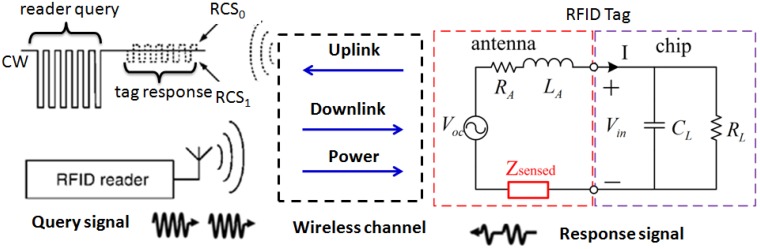
A passive antenna sensor system based on backscatter communication.

The EPC C1G2 standard defines communication between RFID readers and tags in the UHF band [73]. As determined by this protocol, the communication between the readers and tags is reader initiated [74]: The reader first sends out continuous wave (CW) to activate a subset of the tags in its interrogation region and then a query (downlink) asking the tags to respond with their IDs; for the uplink (assuming that the tag IC remains powered), the tag chip alters the reflection coefficient of tag antenna by varying its internal impedance ($Z_{L}=R_{L}+jX_{L}$) so as to enable re-radiation of the readers CW signal (backscatter modulation). The configuration of a passive antenna sensor and system based on this mechanism is shown in Figure 4. In order to maximize the efficiency of WPT, the tag antenna is designed to be conjugately matched with the input impedance of tag chip at its centre frequency. The reflection coefficient, Γ, which accounts for the impedance mismatch between the tag chip $\left(Z_{L}=R_{L}+{\mathrm{jX}}_{L}\right)$ and the tag antenna $\left(Z_{A}=R_{A}+{\mathrm{jX}}_{A}\right)$ with $Z_{A}^{*}$ being its conjugate, is given by:

(1) $$\Gamma =\frac{Z_{L}-Z_{A}^{*}}{Z_{L}+Z_{A}}.$$

The tag’s antenna reflects an amplitude or phase shifted version of the incident signal, where the amount of shift is governed by the antenna’s loading [75]. Assuming the antenna load’s reflection coefficient is $\Gamma_{0}$ or $\Gamma_{1}$ respectively corresponding to bit ‘0’ or bit ‘1’, the captured signal in the reader due to the variation of RCS can be denoted as ${\mathrm{RCS}}_{0}$ or ${\mathrm{RCS}}_{1}$.

The antenna can be a regular antenna fabricated with conventional dielectric materials or coated with functionalized materials in the passive antenna sensor system. The defect directly or indirectly changes the electric property of the antenna sensor, corresponding to its impedance variation. The reader (interrogator) can actively and wirelessly monitor the antenna parameters via wireless channel based on RCS. Then, features are extracted from the backscattered signal and used to detect and characterize the defect. The main purpose of the modulator is therefore to modulate the interrogation signal received by the tag antenna so that the signal backscattered by the tag antenna, i.e., the antenna backscattering, can be separated from the signals backscattered by the surrounding structures, i.e., the structural backscattering [27]. *This is also the major difference between chipped and chipless antenna sensors.*

In order to provide a physical insight about the above interference, the influence of sensing signal via communication and coherent I/Q demodulation was analytically studied with respect to the power and phase measurements in [76]. The derivation procedures are thereby neglected, and the results are directly given out. One can directly measure both power and phase of the received tag signal as follows:

(2) $${\Delta P}_{\mathrm{received}}=\frac{I_{\mathrm{AC}}^{2}+Q_{\mathrm{AC}}^{2}}{Z_{0}},\;\varphi =\arctan\frac{Q_{\mathrm{AC}}}{I_{\mathrm{AC}}},$$

where $I_{\mathrm{AC}}$ and $Q_{\mathrm{AC}}$ are the difference signal in the period *T*, $Z_{0}$ is the input impedance of the receiver, e.g., 50 Ω. One indirect measurable parameter, i.e., the differential RCS or ΔRCS, can be expressed as:

(3) $$\Delta \mathrm{RCS}=\frac{{\Delta P}_{\mathrm{received}}}{P_{\mathrm{in}}G_{R}^{2}}\frac{{\left(4\pi \right)}^{3}d^{4}}{\lambda_{0}^{2}\eta_{p}}.$$

Here, $P_{\mathrm{in}}$ is transmitted power input to the terminal of the reader antenna, $G_{R}$ is the gain of the reader antenna, λ_0_ is the free space wavelength at operating frequency, *d* is the distance between the reader and tag antennas, and $\eta_{p}$ is the polarization mismatch between the two antennas. Assuming the precision (number of bits) of analog-to-digital converter (ADC) is B, the ${\Delta P}_{\mathrm{received}}$, also known as received signal strength indicator (RSSI), at the antenna connection can be given out by [77]:

(4) $$\mathrm{RSSI}=10^{-\frac{G_{\mathrm{rf}}}{10}}\frac{1.2567\times 10^{4}V_{c}^{2}}{\left(2^{2B}\right)R}{\left(\frac{1}{N}\sum_{n=0}^{N-1}\left|Y_{I\;\mathrm{or}\;Q}\left[k,n\right]\right|\right)}^{2}\mathrm{mWatt}.$$

Here, *R* is the input resistance of ADC and $V_{c}$ is the input chip level, $G_{\mathrm{rf}}$ is the analogue gain from antenna connector to ADC input, and $Y_{I\;\mathrm{or}\;Q}\left[k,n\right]$ is the *n*-th sample at the ADC output of *I-* or *Q*-branch within single *k*. Meanwhile, the forward power to activate tag, i.e., $P_{\mathrm{in}}^{\mathrm{to}}$, can be expressed as:

(5) $$P_{\mathrm{in}}^{\mathrm{to}}\left[\Psi \left(\theta ,\;\phi \right)\right]={\left(\frac{4\pi d}{\lambda_{0}}\right)}^{2}\times \frac{P_{\mathrm{th}}}{G_{R}\left(\theta ,\;\phi \right)G_{T}\left[\Psi \left(\theta ,\;\phi \right)\right]\tau \left[\Psi \right]\eta_{p}},$$

where $P_{\mathrm{th}}$ is the minimum incident power needed to activate the tag chip (also called read sensitivity), $G_{T}$ is the gain of the tag antenna, $\tau =1-{\left|\Gamma \right|}^{2}$ is power transmission coefficient, and Ψ represents the defect variable. Here, Γ is the reflection coefficient at the matching state. It is worthy to note that both the $G_{T}$ and τ are dependent on the defect while the former depends on the orientations as well.

Typical passive RFID systems suffer from round-trip path loss; specifically signal-to-noise ratio (SNR) at the receiver drops with the fourth power of reader-to-tag distance, for a two-ray propagation model [78]. Compared with the counterpart of near-field communication (NFC), UHF antenna sensors use standing wave (or evanescent mode) for sensing and propagation mode for communication. The direct usage of the direct measurement quantities, e.g., amplitude [79] and phase [80], is inevitably influenced by the wireless channel. Furthermore, because of the limited receiver’s sensitivity as well as ADC’s resolution, the resolution of the passive antenna sensor systems decreases as an increase of the read range. The challenges and related solutions will be introduced in Section 3.2. At the same time, people are trying to reconstruct the antennas’ parameter by combining several quantities together and obtain the impedance or other robust sensing variables. This part will be introduced in Section 4.3.

### 3.2. Communication-Oriented Issues and Solutions

In a backscatter system, the power received by the tag or backscattered to the reader may drastically vary as a function of tag and reader positions—even when a line-of-sight (LOS) path exists in between. This variation, famous as *small-scale* *fading*, is caused by the constructive and destructive interference of waves scattered from objects in the propagation path.

Passive RFID tags are traditionally assumed to be downlink limited since typical tag sensitivity (downlink) is considerably poorer than reader sensitivity (uplink), because of the stringent power limitations of tag chips. The above highlights an important facet of RFID systems that appears to have been underappreciated in the existing literature — the fundamental *asymmetry* of the uplink and downlink ranges at which information may be reliably communicated [81]. As a result, the small-scale fading effects are more severe than in classical one-way systems [82]. Hence, improving the downlink range for passive tags is a key design objective. With continuing advancements in integrated circuit (IC) technology, future passive tags that operate with reduced power may become uplink limited.

In backscatter communication, the signal received at the reader arrives after traversing two independent paths. On the reader-to-tag downlink, the impinging signal at the tag antenna is the superposition of components from multiple scatterers in the vicinity of the tag. This incident signal is modulated by the tag chip and scattered back to the reader; en route, the backscattered signal encounters another set of scatterers close to the reader. Since the receiver observes the product of two independent small-scale fading effects, the net fading statistics differs from the standard Rayleigh fading, known as the *dyadic backscatter channel* (DBC) model [82].

In general, the backscattered signal is subject to environmental *multipath* en route to the reader that causes both frequency and time selective effects [75]. The uplink symbol rate is sufficiently low, such that we may ignore the impact of any frequency selectivity, i.e., we assume no inter-symbol interference. Typically the physical environment changes slowly over time, so the symbols experience *slow fading* *multipath* conditions.

The channel property will influence the stabilities of measurable parameters directly calculated from the received backscattered signal. For example, RSSI signatures are repeatable (and not merely random noise) when the environment remains unchanged. However, if a change in the environment happens, not all of the frequencies are equally impacted. Instead, a small change in the environment only results in a slight but noticeable change in the shape of the RSSI signature. Furthermore, if an object in the environment is incrementally moved, it will cause a ripple effect [83]. Based on these facts, the shape of the RSSI signature is dependent upon the multipath of the surrounding environments.

For the above reasons, successful backscatter system design requires an understanding of the propagation mechanisms that affect both the power available to the RF tag and backscattered to the reader receiver. Meanwhile, accurate link-budget equations, along with a detailed description of the modulation factor, on-object gain penalties, path-blockage losses, polarization-mismatch losses, impedance-mismatch losses, and small-scale fading losses should be considered ahead [84].

The main performance metric of RFID systems is the reading range or coverage that is defined as the maximum distance between the reader and the tag at which the radiation field from the reader is strong enough to power up the tag and consequently, the backscattered signal from the tag reaches the reader with sufficient power (i.e., with power above the reader’s sensitivity) [85]. For the mono-static configuration, a single antenna is employed to simultaneously transmit the CW signal to power the tag as well as receive the backscattered signal from the tag. For the bi-static configuration, the RFID reader uses two or more co-located or dislocated antennas for separate transmission and reception. It can be found that with proper antenna spacing/orientation, bi-static systems can achieve a larger reading range and a more uniform distribution of tag RSSI in its reading area compared to mono-static systems [86].

As seen in cellular technologies, multi-antenna techniques offer simple and effective solutions that improve the uplink rate or reliability [87]. As a result, multi-antenna techniques in RFID systems have come into the focus of research to overcome the drawback [88]. The most effective way to improve the DBC link reliability is to increase the number of tag antennas. However, this is not practical because the increase in cost and complication of tag antennas is not allowed in most cases. A RFID reader only needs two to four receiving antennas and one transmitting antenna to improve the reliability of uplink; additional receive antennas provide diminishing gains [75].

However, these multi-antenna techniques increase the design complexity of the system. Alternatively, the magnitude of the vector effective lengths associated with tag and reader antennas improves with an increase in their respective antenna gains, which improves both downlink and uplink ranges. Furthermore, it was found that the choice of amplitude-shift-keying (ASK) impedance modulation indices can maximize the operating range as a function of key system parameters notably the tag sensitivity and bit error rate (BER) at the reader [81].

**Figure 5:**
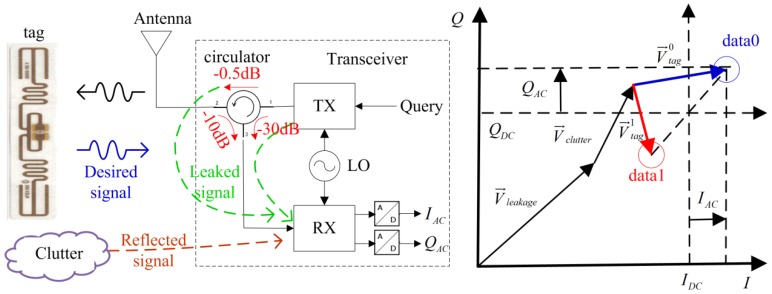
Transceiver architecture and interferences.

The above part describes the sources of uncertainties because of the channel and potential solutions to improve the uplink reliability. A typical interference from wireless channel and transceiver itself is shown in Figure 5. On the other hand, the bottleneck of the passive antenna sensor system is limited by the resolution and sensitivity of the receiver onboard the reader. 

It was shown that the gold encoded messages were received with less error than the Miller-coded ones [89]. This is due to the orthogonality of the symbols as well as the characteristics of the Pseudorandom Noise (PN) codes, that make them less susceptible to environment influences from additive white Gaussian noise (AWGN). Nevertheless, backscatter communications must contend with received noise that is dynamic and colored (because of self-interference) rather than simply static and white (because of thermal noise). In fact, this is an intrinsic limitation of conventional modulated scatterer techniques [90], because colored noise comes from: (1) local-oscillator leakage through a direct down-conversion receiver’s mixer; (2) transmit-receive antenna coupling (in a bi-static reader) or antenna mismatch effects (in a mono-static reader); and (3) the unmodulated carrier reflected from the environment back into the receiver. When compared to a conventional one-way digital radio link, a significant amount of colored phase noise about the RF carrier makes its way through a backscatter receiver’s RF chain.

Careful selection of the bit rate along with the number of *inter-bit transitions* with regards to a reader’s noise spectral characteristics was shown to maximize sensitivity while being mindful of power or energy consumption by the backscatter RF tag [91]. This strategy maximizes the sensitivity of the backscatter modulation while maintaining the tag’s power requirements. The use of *inter-bit transitions* to improve backscatter modulation is not new; for example, the EPC Global C1G2 protocol allows for variations of the *n* = 4, 8, and 16 Miller schemes to increase sensitivity [73]. In addition, using multiple, 45° slant antennas on the RF tag, in conjunction with cross-polarized reader transmitter and receiver antennas, was demonstrated to improve backscatter modulation by reducing the reader’s self-interference [84].

## 4. Sensing-Oriented Issues and Solutions

The measurable parameters of backscatter communication and related channel issues causing unreliability of these parameters are explored in the previous section from communication point of view. From antenna and sensor point of view, the major challenges and state-of-the-art progress about passive antenna sensors and systems are comprehensively investigated in this section from four aspects: defect types and antenna topologies, materials and manufacturing technologies, sensing variable and measurement uncertainties, and feature extraction and characterization.

### 4.1. Defect Types and Antenna Topologies

The design of passive antenna sensors is an interdisciplinary research subject. The antenna sensor can use standing (or evanescent) waves for detection, and this information is transferred to the reader via a propagation wave in a form of RCS but influenced by nearby objects. In fact, an antenna lying parallel (and horizontally polarized) to a conductor will see the impedance of free space on one side, and the (surface) impedance of the conductor on the other side, the latter of which can be written as [92]:

(6) $$Z_{s}=\frac{E_{z}}{H_{y}}=\frac{1+j}{\sigma \delta }=\left(1+j\right)\sqrt{\frac{\omega \mu_{0}}{2\delta }},$$

where σ and δ are the conductivity and skin depth of the conductor, respectively. Here, ω is the angular frequency and $\mu_{0}$ is the permeability in the air (for non-ferrite conductive material). Consequently, the antenna is shorted out by the conductor underneath, leading to a standing wave formed between the antenna and the conductor. Using the method of images and the concept of self- and mutual- impedances, the input impedance of a half-wavelength dipole placed at a height *d* above an infinite conductor is given by [93]:

(7) $$Z_{\mathrm{in}}=Z_{11}-Z_{12}\left(d\right).$$

Meanwhile, the electromagnetic radiation in the far-field is due to the superposition of the antenna current and the image current. Any disturbance in the area between the antenna and the conductor will cause a variation of stored energy, in turn, the resistance and reactance of the antenna’s input impedance. However, the reflection from the conductor and seen by the antenna is polarization, incident angle, and material property dependent [94]. On the one side, this can benefit the defect detection; on the other side, the field distribution in the antenna structure is therefore determined by the antenna mode. This is also the major difference between the antenna sensor and pulsed eddy current (uniform magnetic distribution inside the coil).

To improve the sensitivity as well as the spatial resolution of the antenna sensor, the power scavenged by the tag should be confined into a small region, e.g., using a superlens [95], and re-distributed to properly interact with the defect on the tagged object [96]. The design issues can be listed as follows [97,98]:

- *Metal mountable*: The design of antennas for metal-mountable RFID tags is challenged by a set of limitations: low-profile and conformal structures, to provide good (gain and impedance matching) and reliable operations on conductive platforms of various shapes and sizes.
- *Sensing oriented*: To be successfully turned into sensors, this class of devices should be able to properly detect and characterize the things (e.g., defects on metallic surface), being, for example monotonic, single-valued, and sensitive enough at least in the most critical ranges. As such, the multi-scale, multi-physics of defect phenomena should be properly modeled before the design of antenna sensor in order to guide the selections of antenna topology and operating mode.
- *Balanced performance*: RFID communication and sensing capabilities properly demand for opposite requirements: The tag’s antenna is usually designed to be perfectly matched to the tag chip in a reference condition, e.g., at healthy state, and it undergoes mismatching along with the continuous variation (propagation) of measurand. Therefore, a trade-off between sensing and communication is a challenging task to be tackled.

A remarkable result is that one effective way for an RFID antenna to “sense” the physical status of an object, with negligible degradation of communication, is to convert the change of the external phenomenon into a variation of the input resistance only, while preserving the reactance as stable as possible [99].

The antenna size needs to be reduced down to the scale comparable with defect patch to maximize the sensitivity and resolution. This is also a requirement for easy deployment and less influenced by nearby objects [100]. However, the size reduction causes a poor radiation efficiency (small radiation resistance) and then degrades the communication performance [101]. The sensitivity and communication distance of the system are thus strictly connected to the antenna’s parameters and more to the point, to its quality factor or bandwidth [102]. The quality factor of the antenna represents the ratio of the time-averaged stored energy around the antenna to the radiated (and lossy) power [103]. The high-Q antenna offers a better sensitivity for detection, but can be difficult to be installed on the surface of a metallic structure bacause it is sensitive to the air gap [104]. Furthermore, impedance matching and gain enhancement should be of particular concern in the small antenna sensor design [97].

There are several types of antenna that can meet the requirements, among which patch antenna and its variations are good candidates because of their simplicities (can be easily adapted by researchers from other communities) and controllable field distributions [105]. However, the antenna would be customized for the specific defect in order to optimize its sensing and communication performance. The following part will summarize the advancement of antenna sensors designed for the strain, crack, and corrosion monitoring.

#### 4.1.1. Strain Detection and Characterization

Strain sensors (gauges) are required to detect deformations or structural change occurring in our surrounding infrastructures. For this measurand, the antenna sensor design is to be considered so that the mechanical strain is changed into electrical signals and the electrical signals are transmitted to the reader via RFID technique simultaneously. The strain model addresses two factors affecting the measured sensitivity [46]: (i) the efficiency of mechanical strain transfer from the base structure to the top surface of the RFID antenna sensor; (ii) the substrate dielectric constant change because of strain. Strain is denoted as $\varepsilon =\Delta L/L_{0}$, where ΔL is difference length because of the strain and $L_{0}$ is the initial “zero-strain” length. Typically, the strain is unitless and is expressed in percentage or microstrain (με = ε × 10^−6^).

Deformation changes the electrical length and therefore the resonant frequency of the antenna. The recent evolution of strain measurement using passive antenna sensors can be summarized in Table 1. A meander-line dipole antenna was designed to measure the strain using a controlled shape factor [40]. Nevertheless, the measurable strain level is low because of its poor mechanical property. To fabricate an efficient strain sensor, researchers are in search of a material that can exhibit a large structural change in response to a small applied strain [106]. Therefore, in conjunction with stretchable substrate and conductive materials, the dynamic range of strain level can reach up to 50% by wirelessly monitoring conductor loss resistance variation of a stretchable dipole on fabric substrate [39]. However, the power variation is used as a feature, which is quite susceptible to wireless channel [43]. Consequently, RFS was extracted as a robustness feature using fabric-based embroidered dipole [107]. In addition, an LC resonator was implemented as a chipless sensor [42]. Unfortunately, vector network analyzer (VNA) is required to monitor the RFS.

However, the dipoles antennas are different to be installed with mechanical structures. For this reason, a folded patch antenna was designed to be mounted on metallic surface with a sensitivity of −0.7404 ppm/με [46]. In conjunction with turn-on power measurement, patch antenna was developed to increase the read range to 2.1 m [36]. Nonetheless, the above sensors can only detect one directional strain. This motivates the design of a slotted circular patch antenna, which can be used to monitor the omni-directional strain [41]. Because the operating resonance frequency equals approximately the strain sensitivity (Hz/με) of an antenna sensor, the antenna sensor has relatively low strain sensitivities. Hence, a frequency doubling technique was introduced by utilized two radiation patches working at $f_{0}$ and its second harmonic frequency, $2f_{0}$, respectively in conjunction with a matching network in serially connected in between. Tensile testing showed an enhanced strain sensitivity of −5.232 kHz/με [38]. Nonetheless, the transmitter and receiver should be customized for this purpose.

#### 4.1.2. Crack Detection and Characterization

Despite the fact that engineering components and structures are carefully designed against fatigue failures, more than 50% of mechanical failures are due to the formation of fatigue cracks. The severity of the failure depends on both the crack length and orientation with respect to the loading direction. Transverse cracks are the most common and dangerous cracks because they can reduce a structure’s cross section and therefore lower its structural capacity/integrity. The traditional crack sensing techniques make use of lead wiring for data extraction, the placement and maintenance of large lengths of which is cumbersome and expensive [35]. The development of crack detection and characterization based on passive antenna sensors are summarized in Table 2.

The detection of cracks using coil antenna was studied in early 2003 [108]. Benefiting from low profile and low cost, patch antennas are frequently used for crack sensing. From cavity theory, the sensitive part of such type of antenna can cover its underneath area. Based on the current techniques, most works are focusing on detection of both crack length and orientation, where dual-mode [33] or 2D grid [32,35] was utilized to complete this task.

With a spatial division using multi-patch, a multiplexing antenna sensor was designed to detect a multi-site crack [34]. However, this sensor system is incompatible with the Gen2 standard. It is worthy to mention that the backscattered phase can function as a sensing variable and a sub-mm resolution was achieved in crack width detection using mutual-coupling between two patch antennas [37,100]. The response of backscattered phase is dependent on the wireless channel, making it limited in the in-site monitoring.

#### 4.1.3. Corrosion Detection and Characterization

The interaction of a corrosive environment and tensile stress (e.g., directly applied stresses or in the form of residual stresses) can produce failure in the form of stress corrosion cracking (SCC) in susceptible metallic components [4]. This damage produced is not always obvious to casual inspection, for example, when under paint, so failures can be both unexpected and catastrophic. Thus, early detection of such defects is important in order to have sufficient time for condition-based maintenance. In the early stages of corrosion, a thin layer of oxides appears and causes changes in the conductivity, permittivity and permeability of the metal on the surface [109]. These changes variate with metal type and can be captured by the impedance change of tag antennas. The developments of corrosion detection based on passive antenna sensors are summarized in Table 3.

The corrosion was demonstrated to be detectable using a LF RFID coil antenna by directly monitoring tag’s response in time domain [47]. The feature of peak value is lift-off (or read range) dependent. In order to tackle this issue, a complex impedance measurement was conducted with the help of VNA; meanwhile, a PCA method was utilized to extract a lift-off independent feature [50]. However, the read range of this type of sensor system is limited because of the evanescent coupling. A 3D antenna was designed to be mounted on the metallic surface and the UHF band RFID technique was adopted to transfer the corrosion thickness induced variation by an AID in a 1-m read range [51]. In addition, one chipless antenna was developed to measure the corrosion under water using stub resonator in a 2-m read range; however, the occupied band of the system is not compatible with Gen2 standard and also a VNA was required to obtain the transmission coefficient of S_21_ [48].

As Table 1, Table 2 and Table 3 show, different antenna design and development including configuration can be applied for different sensing applications. The optimal impedance match, gain, and measurement range remain challenges [110]. In addition with the interrogation using narrow-bandwidth RFID technique, the trade-off between sensitivity and dynamic range challenges the antenna-sensor design as well [99]. Meanwhile, the multiple-parameter signature of defects, for example, the crack profile, depth, and location, and the multi-physics procedures in EM modeling and co-simulation [6], complicates the design procedure and optimization including selection of materials.

### 4.2. Materials and Manufacturing Technologies

Simplified processing steps, reduced materials wastage, low fabrication costs, and simple patterning techniques make printing technologies attractive for the cost-effective manufacturing [111]. Such developments are progressing at a fast pace, and demonstrations have been done so far in many areas, including sensors, displays, solar cells, printed batteries, energy harvesters, and capacitors. Above all, additive manufacturing technology [112], substrate materials [113], and conductive materials [114] are three key factors in controlling the cost, chemical, electrical, and mechanical properties for printable sensors.

In general, the printing technology can be categorized into contact and non-contact: the contact-based printing technologies comprise of gravure printing, gravure-offset printing, flexographic printing, and roll-to-roll (R2R) printing; the prominent non-contact printing techniques include screen-printing, slot-die coating, and inkjet printing. Critical limitations of each technology have been highlighted and potential solutions or alternatives have been explored [115]. The R2R fabrication provides the ability to deliver cost-effective technical solutions for sensors and other electronic devices [116]. Among which, the inkjet printing technology received more and more attention because of its simplicity, flexibility, precision, accuracy, high speed, and the capacity to process a wide variety of printing materials. In particular, the specific advantage of this technology is its ability to print a controlled amount of ink, down to 1 picolitre, at high frequency, on almost any type of substrate [117]. However, since a low concentration of the conductive ink is jetted on a substrate, it is difficult to avoid coffee ring effect which results in irregular thickness and low density of the electrode pattern after the ink dries out weakening the resulting electrodes. As a result, relatively low conductivity and low mechanical strength usually can be obtained from jet printing [42]. Understanding in droplet generation, surface chemistry, polymer/substrate selection and process scalability should be exploited [118].

Various flexible substrates can be selected for sensor applications: polymer, semiconductor, organic, ceramics, et al. The circuit board’s tensile strength, allowable temperature of desired flexible substrates, and thickness are likewise significant factors for R2R processing and transferring techniques [119]. Polyimide (PI), which has a high thermal and chemical resistance, is a most widely used flexible substrate [43]. The benefits of using paper as a substrate were also discussed, reporting a good electrical/dielectric performance for frequency up to 1 GHz [120]. In addition, the evolution towards the first integrated RFID-enabled wireless sensor network infrastructure using inkjet-printed electronics technologies on flexible and paper substrates was first reported in [121]. However, the electrical and mechanical properties of RFID chip joints assembled on a flexible substrate need to be considered [122].

The conductive ink plays a key role for printable antennas. Reference [123] reviewed the basic properties of conductive nanomaterials suitable for printed electronics (metal nanoparticles, carbon nanotubes, and graphene), their stabilization in dispersions, formulations of conductive inks, and obtaining conductive patterns by using various sintering methods. Conductive inks generally contain at least one kind of binder to form a continuous film, however, adding insulating binders such as polymeric or siloxane will reduce the ink conductivity [124]. For example, a graphene oxide (GO) assisted liquid-phase exfoliation process was demonstrated for the preparation of high-quality graphene from graphite, which is a little sacrifice of the conductivity, reported as 6.2 × 10^4^ S/m) [125]. The other effective operating parameters on the film formation are surface temperatures, surface energy of the substrate, surface tension, and viscosity of the ink [126]. Furthermore, the evaporation, the film homogeneity, the electrical properties, all rely heavily on ink formulation [117].

The increasing numbers of research articles and demonstrations of printed sensors and electronics in a number of applications reflects the keen interest of researchers to fulfill the promise of large area electronics on flexible substrates through cost-effective printing technologies. Reference [127] investigated for the first time inkjet-printed UHF and microwave circuits fabricated on paper substrates as an approach that aims for a system-level solution for fast and ultra-low-cost mass production. Reference [128] introduced printed electronics through flexible substrates and low-cost fabrication with huge potential for the future integrated smart sensing and network application. Reference [129] demonstrated a prototype printable chipless RFID, which can be easily transferred to plastic, paper, and other material substrates, making it suitable for mass deployment for low-cost items. Reference [58] presented a chipless RFID tag sensor that potential to be printed on flexible laminates for ultra-low cost ubiquitous sensing. Reference [130] discussed new materials and technologies towards emerging flexible sensors, e.g., printing technologies to support low-cost electronic devices for multisensory and monitoring.

The more detailed challenges in printable electronics from materials, technologies, and perspective applications including printed antennas and RFID tags for sensors and integrated smart systems can be found in [131]. Some potential trends are going to be discussed in Section 5.3.

### 4.3. Sensing Variables and Measurement Uncertainties

The RF signals carrying sensed information are backscattered into the wireless channel and passive antenna sensors with the combination of sensing and communication in the system need addressing the RF channel to mitigate path loss and multipath effects [132]. In spite of analogue RFID sensors are gaining increasing attention from academic and industrial domains, their true applicability in the real world is still in question, since it is not clear whether and in which conditions the variation of the measured signals related to the sensing activity may be distinguished from the measurement uncertainties [133].

The defect-induced changes in antenna properties vary the amplitude and phase of a tag’s response and sensing data can be directly or indirectly measured via the RFID reader. Similar to a pulsed eddy current technique [134], the time domain measurement, e.g., transient envelope of tag’s response, has been explored to characterize corrosion in the LF and HF RFID sensor systems [49,50]. This procedure is fast and accurate in near field range but cannot be directly used in UHF band since the extraction of such a transient information needs a high sampling rate, which is not practical to be implemented for a cost-effective receiver. Furthermore, the influence from environments becomes severe because of the scatters in the path of forward and backscattered signals. As coherent receivers can directly provide both amplitude and phase information [135], power and phase become the mostly used sensing data in the UHF band. In particular, to obtain a better consistency and communication range, one of the major challenges for wirelessly monitoring defects is to mitigate influences from the test setup and environment.

For power-based measurements, sensing capability is realized at the expense of the mismatch of the tag antenna impedance and of the decrease in efficiency [136]. Good resolutions in power can be achieved by improving the quantization resolution in the receiver’s ADC, but it is not feasible with low-cost readers. The tradeoff issue between sensing and communication is a major impetus for recent efforts in defect characterization via phase [137]. However, the measurement of phase heavily depends both on the propagation channel and on modulating properties of the tag which can be both frequency- and power-dependent.

The modulation RCS is a concise and effective application of the mature and proven RCS concept, but some challenges arise in its use [138]: The use of modulation RCS in typical indoor deployment environments is complicated by fading that is not studied in the mature radar literature. The tag’s backscatter modulation efficiency of the tag chip is also nonlinear, tending to fall sharply with increasing incident power, so the RCS must also be a function of the incident field strength. Interestingly, benefiting from the power and frequency dependent characteristics of tag chips, a differential RCS measurement significantly improves the sensitivity and increases immunity from the environment [44].

Without prior information about the tag-reader mutual position, multiple measurements can be applied for separation and reductions of multiple influences and also improving the repeatability. The drawback using power measurement has been partially solved by combining the forward power and backscattered power and a sensing variable named AID was invented for this purpose [18]. In fact, AID is only related to impedance rather than antenna gain [100].

By means of ad-hoc test-beds, it was demonstrated that backscattered power, or RSSI, exhibits a combined uncertainty ranging from 0.5 to 2 dB, with a deep dependence on the measurement instrumentation, which implies only sensors with large dynamic ranges could be used in real applications [136]. Meanwhile, for a confidence level of 95%, the measurement uncertainty on the ΔRCS is calculated and found to be 2.27 dB or 29.8% [139]. Even without recalibrating demodulated backscatter from a spectrum analyzer, AID would have the uncertainty of an order of 1 dB, which has a 1-dB improvement compared with traditional RCS measurement [140]. AID can be measured to within 0.5 dB of absolute uncertainty with calibrated modulation power measurements [17]. This propagates to about the same uncertainty in estimates of the minimum bound for backscattered power. Therefore, compared with $P_{\mathrm{in}}^{\mathrm{to}}$, RSSI, and *ΔRCS*, AID is preferred among power metrics in terms of repeatability [136].

### 4.4. Feature Extraction and Characterization

The process to extract features from RCS and related parameters is an inverse problem [141,142]. It is of paramount importance to mitigate multiple influences to get robust sensing information from the RFID sensor system. Several interferences including sample surface geometry, multiple scattering because of nearby objects, and reader distance between the tag and the reader are mixed and thus need to be separated. The antenna sensor can sense the defect through the extracted feature from sensing variables, but the interferences change the impedance and radiation pattern of the antenna and therefore force the change in sensing variables as well [51]. A robust sensing variable can be selected accordingly as in Section 4.3. Feature extraction method should be utilized to for solve the ill-posed problem and then carefully estimate the defect.

As seen in Section 4.1, RFS is widely used to characterize defects because of its simplicity and robustness [27]. However, the feature of RFS only considers the local structure of the data manifold and thus could lose important information existing in the global structure of the given data [143]. More importantly, this feature challenges the antenna design and installation because the high-quality factor is required [144].

As known to us, the characteristic mode analysis is a method used in electromagnetics, which gives insight into the potential resonant characteristics of a structure by finding and examining the inherent modes of the structure [145]. The input admittance of the antenna at a feed point m can be expressed as a summation of the modal admittances [146]:

(8) $$Y_{\mathrm{in}}\left[m\right]=\sum_{n}\frac{J_{n}^{2}\left[m\right]}{1+\lambda_{n}^{2}}\left(1-{j\lambda }_{n}\right).$$

Physically, the eigenvalue $\lambda_{n}$ represents the net stored energy of the mode and take real values from −∞ to +∞, with negative and positive values representing net electric and magnetic energy storage, respectively. The characteristic currents $J_{n}$ are the real-valued eigencurrents (eigenvectors) of the mode on the structure and give rise to the modal radiation patterns and other field quantities [147]. The resulting eigenvalues and eigenvectors, i.e., $\lambda_{n}$ and $J_{n}$, are frequency dependent but independent of the excitation.

The above insight can build a bridge between impedance and extracted feature [148]. For this reason, pattern recognition methods can be used for feature extraction. In particular, as typical supervised learning algorithms, PCA and independent component analysis (ICA) are broadly investigated to be a feature extractor because of their ability to find the eigenvector that dominates the variance and statistically separate the desired signal from interferences. At the same time, the experienced limitations in term of uncertainties and achievable resolutions suggest a potential usage of low-cost analogue RFID sensors for providing a few-level sets of things. As a result, the analogue RFID sensing can be hence addressed as a classification problem and accordingly well assessed classification algorithms, like the PCA could be applied to multiple indicators to improve the resolution and/or the detection robustness [133]. Enhancing both the sensitivity and robustness was demonstrated for corrosion detection and characterization in conjunction with PCA method [51].

## 5. Future Trends and Perspectives

The two sections above summarize the challenges and solutions for the practical applications of passive antenna sensors and systems. Some technical limitations which remain unresolved are studied in conjunction with emerging techniques to expand the applications of passive antenna sensors and systems. For this purpose, the future trends are categorized into three directions with more detailed perspectives: (1) networking and standardization: array or tag-tag coupling for improving coverage, integration with UWB technology for data-intensive applications, standardization for integration of sensing capability, evolution of Wireless Integrated Sensing Platforms (WISPs) for reduction of power consumption and integrations with more external sensors, integration with WSNs or developed into RFID sensor network (RSN), and integration with narrow-band IoT; (2) more ubiquitous and adaptable: integration with more chip-embeddable sensors, automatic impedance matching and digitalization of RSSI, analogue memory with function materials, wearable electronics for healthcare applications; (3) more simple and reliable: software defined radio (SDR) for much simple and low-cost readers, chipless antenna with variable coding mechanism, harsh environment monitoring. Based on the trends, previous publications, and long-term vision, some remarks are suggested, in particular, for the potential applications of the systems in the UK.

### 5.1. Integration and Standardization

The rapid evolution of large-area electronics printing technologies, e.g., inkjet printing, has enhanced the development of low-cost RFID-enabled sensors as well as accelerated their high granularity deployment in large scale structures. Tag and tag communication [149], grid issues [150,151,152], e.g., granularity and cross-talk, might be considered or utilized to enhance the resolution, coverage, and detection of the inter-tag defects.

Integrating with UWB technology is a promising solution for next generation RFID systems to overcome most of the limitations of the current narrow bandwidth RFID technology such as: low-data rate, reduced area coverage, insufficient ranging resolution for accurate localization, sensitivity to interference, and scarce multiple-access capability [153,154]. The maturation of passive low-cost RFID tag technology has made it a viable candidate for scenarios where short-range, low-rate links suffice. A recent innovative trend centres on re-engineering passive RFID tags towards WSN applications, i.e. to more data-intensive applications rather than tag identification applications [155,156,157]. The tasks involved in the integration of WSNs and RFIDs are to tackle issues of energy conservation, real-time performance, data cleaning and filtering, localization, anti-collision, and authentication [158].

The success of IoT depends on standardization, which provides interoperability, compatibility, reliability, and effective operations on a global scale [17]. There are already several standards such as the International Organization for Standardization (ISO) and Electronic Product Code (EPC) Global, which allow for the simultaneous interrogation of multiple tags with a low data-collision probability for a variety of environments and tag configurations [73]. The anticipated higher data rates for sensor nets will exacerbate tag collisions on the uplink with existing protocols; future RFID networks are thus likely to be uplink limited, based on this consideration. Compressive sensing (CS) can be applied to reduce the ID search space and thus read more tags in a shorter time [159]. This technology can also be utilized to reduce the stringent data rate requirement enabled by ubiquitous computing in the tag side [160]. On the other hand, as deployments scale to larger tag populations requiring in turn many more readers in a given area, the likelihood of reader collisions (inability by tags to decode reader commands) on the downlink will also increase (for a given frequency band or number of channels) [74].

As the number of users, data volume, and range of sensor systems grow, passive backscatter- based networks will require improved links and power efficiencies, thereby opening a new set of challenges for RFID system designers at all (circuit, device, communication link and network stack) levels [82]. Extending the chip’s interface capabilities to a sensor is straightforward. An example of a passive sensor platform with power harvesting ability is the WISP [161]. In addition to the basic identification functions of conventional tags, WISP is equipped with sensors connected to a microcontroller unit, thus providing sensing and computing capabilities [162]. Moreover, it is powered and read by standard Gen2 readers [163]. Though extremely flexible and versatile, the WISP solution is, of course, more expensive than traditional passive RFID tags and has limitations in terms of read range, that is almost 3 m [164].

The integration of RFIDs and WSNs will increase their combined data reporting capabilities, e.g., context-aware services [165]. But the standardization activities in this area remain unclear since RFID and sensors have been traditionally covered by different standardization bodies [166]. Seamless communication can thus be problematic in a multi-entity business model such as supply chain logistics if there is no one standard which is agreed upon and if one or more of the partners in the chain do not have the infrastructure in place to interrogate these sensing units [11]. This standardization is a must go area in the integration of RFIDs with WSNs, or developing a RSNs [167].

Besides, narrow-band IoT (NB-IoT) is a new radio technology standard that has been developed to enable a wide range of devices and things to be connected using long-term evolution (LTE) system [168]. The standardization of release 13 has been completed on June 2016 by the 3rd Generation Partnership Project (3GPP), which is one of a range of Mobile IoT (MIoT) technologies [169]. The transmitted power of the reader is limited to 23 dBm for communicating with cellular base-station [170]. Therefore, the design challenge is both the hardware, software, and firmware of the reader. More specifically, the antenna gain and bandwidth as well as the data collection, processing, and transmission latency play a strategic role for the integration and real-time monitoring.

### 5.2. More Ubiquitous and Adaptable

As RFID becomes more prevalent, growing economies of scale will enable the integration of environmental sensors with tags reporting on a wide range of conditions. Great efforts are dedicated to the development of RFID chips with integrated sensors where the sensor is powered by the RFID reader signal. This fascinating solution imposes strict constraints on the sensor, which should be both energy efficient and chip-embeddable [162]. Usually, only a few kinds of sensors satisfy such requirements: temperature, light, and pressure sensors are the most common [171,172,173].

The automatic impedance matching (self-tuning) is capable of compensating for the influences of changing objects close to the antenna, thus achieving a constant high reading performance [174]. This can be applied to improve the matching performance of tag antenna and thus to tackle the tradeoff between sensing and communication. For example, a Magnus S Sensor chip supplied by RFMicron can operate at temperatures ranging from −40 °C to +85 °C and it consists of a sensor code and on-chip RSSI code [175]. Alternatively, the sensed quantity can be obtained with a self-tuning module which contains a tuning element to compensate for the changed impedance because of tagged object (defect). The digitalized information can reconstruct the RSSI or impedance of tag antenna and consequently alleviate the influence of the channel in the backscattered communication [176]. Therefore, the reliability and measurement uncertainty of passive antenna sensors and systems can be greatly improved.

Compared with battery-powered sensors, passive antenna sensors have drawbacks in terms of sensing range, lack of time history data storage, and non-real-time data communication [11,177]. More function materials can be embedded into antenna sensors, to make them smarter, e.g., Shape Memory Alloys (SMA) for memorizing a violation in history [178]. A single event logging functionality by means of direct integration of a printed 1-bit write-once-read-many (WORM) memory into the antenna structure was developed to be a humidity sensor, whose value can be read out at a later occasion since the WORM memory records an event by changing its state [57]. Chemical sintering of silver metal nanoparticles and the deliquescence phenomenon of salts were exploited to monitoring the exceedance of a r.h. threshold without the need of a permanent electric energy supply [179].

Wearable electronics have received an extensive interest because of the great potential of future wireless body area networks (WBANs) [180], which can be used to monitor the movement [181] or vital signs of human being [182]. In particular, there is a growing demand for cost-effective textile antennas that can endure stretching and moisture for future WBAN and sensing systems [183]. Additive manufacturing provides the foundation for wearable applications, as it has the capacity to integrate with soft and stretchable materials [184,185,186,187,188]. Electrocardiograms (ECGs) are one of the most common forms of non-invasive diagnostics. The wiring harness connecting a patient to an external ECG monitor poses a significant problem for monitoring ambulatory activities and in long-term monitoring, because of the potential for discomfort and impeded movement. For this reason, a passive wireless multichannel telemetry device capable of transmitting an ECG to an external system was presented [189]. A small-size epidermal RFID thermometer, suitable for the direct placement over the skin, was developed, satisfying the target value for standard thermometers (ear 0.2 °C, underarm 0.5 °C) after uniform recalibration [66]. The cost, size and ruggedness advantages shows that passive sensors can offer some potential applications for such devices, e.g., for pills and implanted biomedical sensors.

### 5.3. More Simple and Reliable

The issue is of interest for existing real-world systems for the following question: How far can the tag signal be heard and correctly decoded in a real environment? It has obvious implications for privacy and security of current RFID deployments but is also an important input for the design of novel distributed systems based on low-cost Rx-only devices [190]. The reader design is based on COTS components-notably the Universal Software Radio Peripheral (USRP) and the GNU Radio signal processing toolkit. The USRP is a low-cost, general purpose RF front end for SDR development that interfaces with a standard PC via USB, with nearly signal processing being performed on the host using GNU Radio software [74]. The usage of this platform makes the access of physical layer and integration with other spectrum easy [191].

Chipless RFID tags and systems are not new [192]. A chipless RFID tag can be fabricated on flexible substrates by printing technologies using conductive inks because it does not include bulky chips but only a metal pattern as an antenna so that fabrication cost goes much further down [193]. Meanwhile, printability of the tags on stretchable substrates is also desired to enable the RFID tag to be conformable to any surface [42]. Sensor-based chipless structure rolling as a monolithic construction can be mounted (or implanted) on safety critical structures as a smart-skin. A major challenge for the chipless tags is the generation of UID. The frequency division, time division, spatial division, even phase division can be used to generate the ID, each of which has its own advantages and disadvantages. Furthermore, 2D structures (patterns), e.g., meta-surface [194], frequency selective surface (FSS), even absorber based chipless antenna and their printing manufacturing are developed for this purpose. However, while some bits of the ID code are used to transmit the value of the sensed parameter [195], the performance for RCS measurement is dependent on several factors, e.g., environment, polarization [196], calibration. These limit the achievable read range and reliability [197]. Above all, the metal-mountable design and anti-collision for multiple chipless RFID tags are still big challenges. In addition to the advantages of chipped sensors, this type of sensor has potential *for future integrated smart multi-sensing and monitoring because of its ultra-low-cost and ability to work in extreme environments.*

The sensing ability of hazardous and flammable substances in the environment has received much attention because of the demands of various application fields, such as disaster prevention, home automation, healthcare, and advanced traceability systems [198]. At the same time, passive antenna sensor technology at an absence of electronic device allows for the inspection and monitoring in areas that are dangerous for humans to carry out activities, for example, energy systems (e.g., oil and gas, nuclear plants, off-shore renewables, etc.) and infrastructure (e.g., bridges, roads, and rail). Based on the fact that the dielectric constant of a ceramic material monotonically increases versus temperature, a chipless RFID tag was applied to design temperature sensor reliably working in harsh environment, e.g., inside the combustion chamber of gas turbines with a temperature as high as 1000 °C [199]. Using a high-Q Zr0.8Sn0.2TiO4 (ZST) dielectric resonator and without patterned electrodes or metallization, a sensitivity of −4500 ppm for the resonant frequency shift was achieved at the range of 200–700 °C in a 1.2-m distance [200].

### 5.4. UK Highlights

RFID-based sensing and monitoring combined with printed electronic devices is leading the way over traditional sensors to have great potential for ground-breaking sensing and monitoring for infrastructures in extreme environments [201], and intelligent society including intelligent packaging [193] and wearable ‘smart’ electronic devices for e-healthcare of ageing people at senior centre, hospital, or home [181]. The passive antenna sensors, in fact, is a new technique and have great potentials to be developed into permanent embedded sensors for ageing infrastructure and life extension: e.g., railway track [202], power plant [203], aircraft [204], oil & gas structures [205] and pipelines [206], where reliable and accurate defect assessment and continuous monitoring is thereby required to provide significant safety and economic benefits. More applications can be found in [207]. This needs interdisciplinary efforts, for example, material science: function material and properties, electronic engineering: electronics and circuit, microwave, information science: networking and convex optimization, and processes: machinery and fabrication. The related researches across the UK for these types of sensor are listed into several categories shown in Table 4.

The current focus in additive antenna fabrication has been mainly to use metallic components as the conductive element. This brings some limitations, including the antenna quickly becoming corroded and oxidized and especially the high material costs. One of the most potential solutions lies on utilizing novel carbon-based nanomaterials, such as graphene, in RFID-based wireless components [124]. In addition to good printability, graphene inks offer great eco-friendly aspects and low material costs. Also, as graphene properties change accordingly with humidity and mechanical stress, by using graphene in RFID antennas, the changes in properties can be exploited into changes in wirelessly measurable parameters [183], providing a huge potential for wearable sensor applications.

Above these, many new opportunities are emerging in the UK. For example, carbon-fiber reinforced plastics (CFRP) composites have been widely used in aerospace, shipping, and automotive structural applications, thanks to their superior stiffness and strength characteristics, fatigue and corrosion resistance. However, because of continuous use and exposure to events, the performance of composite structures can be easily affected in terms of local defects like fiber breakage, resin rich zones, delamination, and impact [208].

In order to cope with such a vision, the unique properties of defects on metal and CFRP should be detailed in order to make the sensor reliable. For example, the electrical conductivity of the CFRP is anisotropic and much lower than the metal counterpart, which is a major concern using EM method; while reduction of the permeability is another concern for corrosion. Furthermore, downstream of overall collaboration, based on the judge to integrated innovation to market demand is not easy for universities alone, and organizations such as RCNDE and TWI can find their benefits. Whilst each industrial sector has its specific requirements, there is a large overlap between sector requirements that can usefully steer and direct research programs through the collective RCNDE industrial membership [71]. In addition to the funding from different technology readiness levels, e.g., EPSRC, TSB, private sectors from industries and third parties, the disruptive innovation in the universities, however, can happen with enough sharing information from both industrial partners (e.g., typical samples) and other institutions (open access database with other NDT&E or SHM methods) [225].

Above all, the world is in the era of IoTs. The UK possesses strengths in both the materials, e.g., graphene, and applications, e.g., nuclear plants, off-shore renewables, railway tracks, e-healthcare, which are at the start and end of the industrial chain. Therefore, we have both the academic and market values for the passive antenna sensors and systems. In addition to the highlighted foreground, future directions, and difference of the UK with the rest of the world, the UK may focus on the robust antenna sensor design (e.g., automatic impedance matching, sensitivity, and gain enhancement), low-cost printing and applications as future leading directions.

## 6. Conclusions

This paper has presented an overview of the progress made in the applications of passive antenna sensors and systems based on RFID technology, particularly for defect detection in metals for SHM. The related issues have been summarized into four main categories: defect type, antenna sensor, measurement strategy, and feature extraction. The challenges, reasons, and state-of-the-art progress for each part have been presented in detail, which offers a comprehensive understanding for problems and guidelines in this area. Emerging techniques for the implementation of passive antenna sensors and systems to make them more adaptable and reliable have also been discussed. In particular, some suggestions on the future R & D for potential health monitoring in the UK have been provided.

The passive antenna sensors offer an excellent potential technical solution for future SHM applications in terms of sensing, communication, location and identification. Several challenges need to be solved before bring this idea into practice. Of course, this type of sensors, can be expanded to other monitoring applications, e.g., environmental monitoring, personal healthcare. The issues and considerations of this review can also be applied to wide ranges of RFID sensor systems and applications beyond SHM.

## Figures and Tables

**Table:** 

Journal Titles	Count	% of 442
IEEE Sensors Journal	53	11.99
Sensors	24	5.43
IEEE Transactions on Antennas and Propagation	23	5.20
Sensors and Actuators B-Chemical	16	3.62
IEEE Transactions on Microwave Theory and Techniques	14	3.17
IEEE Journal of Solid-State Circuits	12	2.71
Sensors and Actuators A-Physical	9	2.04
IEEE Transactions on Instrumentation and Measurement	9	2.04
(**b**)		

**Table:** 

Countries/Territories	Count	% of 442	Organization-UK	Count
USA	178	40.27	University of Cambridge	7
P. R. China	71	16.06	Imperial College London	6
Italy	57	12.90	Newcastle University	5
Spain	52	11.76	University of Manchester	4
Germany	38	8.60	University of Bristol	4
South Korea	27	6.11	University of Kent	3
France	27	6.11	Middlesex University	2
Japan	22	4.98	Queen Mary University of London	2
(**c**)				

**Table 1:** List of RFID tag antenna sensors for strain detection and characterization.

Sensing Principle (Antenna Type)	Sensing Variable	Feature	Pros.	Cons.	Refs.
Conductor loss resistance (stretchable dipole on fabric substrate)	Backscattered power at turn on threshold	Power variation	Strain level up to 50%;Sensitivity can be modified by conductive material; Read range: 1.5 m	Power is susceptible to wireless channel	[39]
Deformation of shape factor (meander line dipole)	Backscattered power	Power variation	Sensitivity: 16%; Read range: 0.6 m	Strain level up to 6% (poor-elastic conductor leads to small yield point)	[40]
Coupling (slotted circular patch)	Reflection coefficient (S_11_)	RFS	Omni-directional strain sensing	VNA is required; Not compatible with Gen2 regulation	[41]
Electrical length (fabric-based embroidered dipole)	Dual-interrogation-mode (read range/RCS)	RFS	Strain level up to 16%;Sensitivity: 0.66 parts per million (ppm)/με	Read range: 20 cm; Need calibration; Dedicated receiver	[107]
Elastic deformation (patch)	Turn on power	RFS	Read range: 2.1 m; Can be mounted on metallic surface	Sensitivity: 0.7907 ppm/με	[36]
LC resonator (PDMS substrate stamped with sliver nano ink)	Reflection coefficient (S_11_)	RFS	Strain level up to 7%; Chipless; Good mechanical	Sensitivity: 0.51 ppm/με; Read range: 20 cm; VNA is required; Dedicated receiver	[42]
Deformation of slot width (dipole on PDMS substrate with stretchable conducting Lycra fabric containing silver threads)	Turn on power	Power variation	Sensitivity: strains of up to 10% causing transmit power differences of about 4 dB; Read range: 1.6 m; Good repeatability	Power is susceptible to wireless channel	[43]
Elastic deformation (folded patch)	Turn on power	RFS	Can be mounted on metallic surface	Sensitivity: −0.7404 ppm/με; Read range: 30 cm	[46]
Elastic deformation (dual patches)	RCS	RFS	Sensitivity: −5.232 kHz/με	Not compatible with Gen2 regulation Dedicated receiver	[38]

**Table 2:** List of RFID tag antenna sensors for crack detection and characterization.

Measurand	Sensing Principle (Antenna Type)	Sensing Variable	Feature	Pros.	Cons.	Refs.
Crack depth	Inductive (coil)	Potential drop	Voltage ratio	Resolution: 0.5 mm in depth	Location dependent; VNA is required	[108]
Crack (length) growth and orientation detection	2D grid (meander line dipole)	Reflectometry	Time difference of arrival	Chipless; Large dynamic range	VNA is required	[32]
Crack (length) growth and orientation detection	Mode orthogonality (dual-resonant patch)	S11	RFS	Resolution: sub-mm; Large dynamic range	VNA is required; not compatible with Gen2 regulation	[33]
Crack (length) growth and orientation detection	Spatial division (dual-resonant patch)	Backscattered power	Power variation	Multi-site crack	Dedicated receiver	[34]
Crack (length) growth and orientation detection	2D Grid (dipole)	Backscattered power	Power variation	Read range: 1 m	Power is susceptible to wireless channel	[35]
Fatigue crack	Deformation (patch)	Turn on power	RFS	Read range: 2.1 m	Large antenna size	[36]
Crack (width) growth	Mutual coupling (patch antenna array)	Backscattered phase	Phase shift	Sub-mm resolution; Platform tolerance	Crack position should be known prior;	[37,100]

**Table 3:** List of RFID tag antenna sensors for corrosion detection and characterization.

Sensing Principle (Antenna Type)	Sensing Variable	Feature	Pros	Cons	Refs.
Inductive coupling (coil)	Envelope	Peak value	Fast	Read range: 3 cm; Lift-off dependent	[47,49]
Inductive coupling (coil)	Complex impedance	principal component analysis (PCA)	Lift-off independent	Read range: 2.5 cm; VNA is required	[50]
Capacitive coupling (3D antenna)	Analogue identifier (AID)	PCA	Read range: 1 m; Wireless channel independent	Antenna profile: 1.6 cm	[51]
Stub resonator (patch antenna)	Transmission coefficient (S_21_)	RFS	Chipless; Read range: 2 m	Influence from immersed water; Not compatible with Gen2 regulation; VNA is required	[48]

**Table 4:** Related researches for the Universities in the UK.

Areas and Focus	Universities
Materials and graphene	University of Cambridge [209] University of Manchester [210]
Wireless power transmission	Imperial College Condon [211,212,213]
Antennas	Queen Mary University London [214,215]
Channel and communication	Queen Mary University London [216,217]
Security and privacy	University of Bristol [218]
Sensors and systems	Newcastle University [49,50,51]
Manufacturing	Loughborough University [219] University of Kent [220]
Smart objects applications	Auto-ID Labs at University of Cambridge [221]
WBAN for e-health monitoring applications	Queen Mary University London [222,223,224]
